# Correction: DNA replication protein Cdc45 directly interacts with PCNA via its PIP box in *Leishmania donovani* and the Cdc45 PIP box is essential for cell survival

**DOI:** 10.1371/journal.ppat.1011303

**Published:** 2023-03-31

**Authors:** Aarti Yadav, Varshni Sharma, Jyoti Pal, Pallavi Gulati, Manisha Goel, Udita Chandra, Neha Bansal, Swati Saha

S1 File was accidentally omitted from the Supporting Information files at the time of production. It can now be viewed below.

## Supporting information

S1 File Supplementary methods. Contains descriptions of various methods used in the study.
(DOCX)
