# Supplementary material for: DNA replication protein Cdc45 directly interacts with PCNA via its PIP box in Leishmania donovani and the Cdc45 PIP box is essential for cell survival
Source: PLoS Pathog. 2020 May 15;16(5):e1008190. doi: 10.1371/journal.ppat.1008190 (PMC7255605; doi:10.1371/journal.ppat.1008190)
Supplement: S1 Table — The data from three independent experiments have been tabulated. For each time point, the percentage of gated cells at different cell cycle stages (G1, S and G2/M) have been indicated for each cell type (cdc45+/+/+, cdc45-/+/+ and cdc45-/-/+), along with averages and standard deviations. Statistical significance of differences between knockout cells and wild type cells were determined using student's t-test. *** p < 0.0005, ** p < 0.005, * p < 0.05. (PDF) [file ppat.1008190.s001.pdf]

S1 Table: Comparative analysis of flow cytometry profiles of *cdc45* knockout lines.

TABLE S1

***cdc45*+/+/+**

	Exp 1	Exp 2	Exp 3	Average	S.D
**Log**					
**G1**	56.09				
**S**	16.37				
**G2/M**	25.2				
**HU**					
**G1**	67.85	70.18	67.79	68.61	1.363
**S**	16.86	18.89	17.47	17.74	1.042
**G2/M**	14.24	9.97	14.19	12.80	2.451
**3h R**					
**G1**	12.01	15.42	15.75	14.39	2.071
**S**	59.29	56.74	54.15	56.73	2.570
**G2/M**	28.27	25.59	29.25	27.70	1.895
**4.5h R**					
**G1**	9.15	6.82	10.96	8.98	2.075
**S**	35.28	38.93	32.56	35.59	3.196
**G2/M**	54.92	53.9	56.63	55.15	1.379
**6.5h R**					
**G1**	9.5	10.55	11.36	10.47	0.933
**S**	20.95	28.92	29.24	26.37	4.697
**G2/M**	66.48	60.53	59.38	62.13	3.811
**8h R**					
**G1**	32.22	32.38	33.36	32.65	0.617
**S**	21.42	21.68	20.85	21.32	0.425
**G2/M**	46.53	45.62	45.5	45.88	0.563
**10h R**					
**G1**	43.27	44.52	44.42	44.07	0.695
**S**	26	24.33	24.75	25.03	0.869
**G2/M**	28.85	30.19	29.77	29.60	0.685
**HU**					
**G1**	58.49	54.27	54.35	55.70	2.414
**S**	23.87	29.15	26.37	26.46	2.641
**G2/M**	16.73	15.16	18.03	16.64	1.437
**12h R**					
**G1**	58.31	45.88	46.75	50.31	6.939
**S**	13.58	21.56	20.48	18.54	4.329
**G2/M**	26.98	31.17	31.12	29.76	2.405
**14h R**					
**G1**	57.7	58.68	58.34	58.24	0.498
**S**	15.99	15.64	14.24	15.29	0.926
**G2/M**	24.83	24.47	25.92	25.07	0.755
**16h R**					
**G1**	54.22	59.07	56.47	56.59	2.427
**S**	16.33	15.71	13.53	15.19	1.471
**G2/M**	27.65	23.89	28.34	26.63	2.395

***cdc45*-/+/+**

						Comparison to *cdc45*+/+/+	
	Exp 1	Exp 2	Exp 3	Average	S.D	p value	Significance
**Log**							
**G1**	65.64						
**S**	15.69						
**G2/M**	17.29						
**HU**							
**G1**	74.31	62.94	73.14	70.13	6.254	0.701323	ns
**S**	11.92	18.7	10.86	13.83	4.254	0.19659	ns
**G2/M**	13.08	17.11	15.27	15.15	2.018	0.268472	ns
**3h R**							
**G1**	11.41	13.41	14.6	13.14	1.612	0.454588	ns
**S**	60.13	59.44	60.31	59.96	0.459	0.098521	ns
**G2/M**	27.82	26.26	24.66	26.25	1.580	0.364268	ns
**4.5h R**							
**G1**	9.54	12.8	10.65	11.00	1.657	0.258133	ns
**S**	56.68	54.98	50.92	54.19	2.959	0.001781	**
**G2/M**	33.17	31.76	38.24	34.39	3.408	0.000612	**
**6.5h R**							
**G1**	8.14	11.08	12.81	10.68	2.361	0.894677	ns
**S**	35.54	31.6	34.24	33.79	2.008	0.065545	ns
**G2/M**	57.2	57.4	52.87	55.82	2.560	0.076031	ns
**8h R**							
**G1**	18.99	25.75	18.04	20.93	4.204	0.008775	*
**S**	20.69	22.46	29.32	24.16	4.558	0.343102	ns
**G2/M**	58.43	51.61	52.46	54.17	3.717	0.01883	*
**10h R**							
**G1**	34.75	38.65	31	34.80	3.825	0.014495	*
**S**	24.34	19.77	24.08	22.73	2.567	0.216014	ns
**G2/M**	41.48	41.07	44.34	42.30	1.781	0.000324	***
**HU**							
**G1**	64.23	62.92	63.43	63.53	0.660	0.005635	**
**S**	17.94	19.05	18.46	18.48	0.555	0.006881	*
**G2/M**	11.11	16.84	16.94	14.96	3.337	0.468941	ns
**12h R**							
**G1**	56.47	45.39	45.81	49.22	6.279	0.849966	ns
**S**	13.53	22.18	21.7	19.14	4.861	0.881554	ns
**G2/M**	28.34	30.23	30.59	29.72	1.209	0.982305	ns
**14h R**							
**G1**	56.8	56.85	56.6	56.75	0.132	0.007425	*
**S**	13.61	14.07	14.09	13.92	0.272	0.07022	ns
**G2/M**	27.72	27.29	27.14	27.38	0.301	0.007915	*
**16h R**							
**G1**	56.88	58.48	57.33	57.56	0.825	0.545387	ns
**S**	13.76	14.08	13.91	13.92	0.160	0.210259	ns
**G2/M**	27.3	25.99	26.63	26.64	0.655	0.993024	ns

***cdc45*-/-/+**

						Comparison to *cdc45*+/+/+	
	Exp 1	Exp 2	Exp 3	Average	S.D	p value	Significance
**Log**							
**G1**	65.37						
**S**	15.01						
**G2/M**	18.41						
**HU**							
**G1**	69.63	63.1	66.85	66.53	3.277	0.367469	ns
**S**	16.33	18.67	17.63	17.54	1.172	0.838676	ns
**G2/M**	12.57	17.19	14.9	14.89	2.310	0.343653	ns
**3h R**							
**G1**	66.75	59.3	65.85	63.97	4.066	0.000047	***
**S**	17.57	21.73	18.15	19.15	2.253	0.000045	***
**G2/M**	14.2	17.83	15.48	15.84	1.841	0.001472	**
**4.5h R**							
**G1**	21.31	22.62	22.07	22.00	0.658	0.000490	***
**S**	49.98	48.27	47.06	48.44	1.467	0.003194	**
**G2/M**	27.7	28.14	30.12	28.65	1.289	0.000017	***
**6.5h R**							
**G1**	18.56	18.42	16.79	17.92	0.984	0.000679	**
**S**	46.72	42.18	42.82	43.91	2.457	0.004592	**
**G2/M**	34.21	38.99	37.79	37.00	2.487	0.000667	**
**8h R**							
**G1**	10.47	13.77	12.61	12.28	1.674	0.000039	***
**S**	44.4	42.22	40.04	42.22	2.180	0.000083	***
**G2/M**	44.98	43.45	47.32	45.25	1.949	0.617423	ns
**10h R**							
**G1**	8.39	12.97	12.5	11.29	2.520	0.000027	***
**S**	39.85	34.09	39.36	37.77	3.193	0.002629	**
**G2/M**	52.13	53.05	47.9	51.03	2.747	0.000196	***
**HU**							
**G1**	64.59	64.44	63.43	64.15	0.631	0.004215	**
**S**	19.35	19.6	18.46	19.14	0.599	0.009409	*
**G2/M**	14.96	14.78	16.94	15.56	1.198	0.374054	ns
**12h R**							
**G1**	24.83	25.92	24.84	25.20	0.626	0.003353	**
**S**	33.28	30.73	27.64	30.55	2.824	0.015808	*
**G2/M**	40.08	42.18	45.7	42.65	2.840	0.003876	**
**14h R**							
**G1**	30.05	30.82	29.23	30.03	0.795	0.000001	***
**S**	32.74	31.78	29	31.17	1.942	0.000216	***
**G2/M**	34.98	35.03	38.96	36.32	2.284	0.001262	**
**16h R**							
**G1**	45.9	46.55	45.38	45.94	0.586	0.001794	**
**S**	24.05	23.51	22.18	23.25	0.962	0.001363	**
**G2/M**	28.16	27.85	30.54	28.85	1.472	0.242576	ns
