# Supplementary figures and images for: DNA replication protein Cdc45 directly interacts with PCNA via its PIP box in Leishmania donovani and the Cdc45 PIP box is essential for cell survival
Source: PLoS Pathog. 2020 May 15;16(5):e1008190. doi: 10.1371/journal.ppat.1008190 (PMC7255605; doi:10.1371/journal.ppat.1008190)

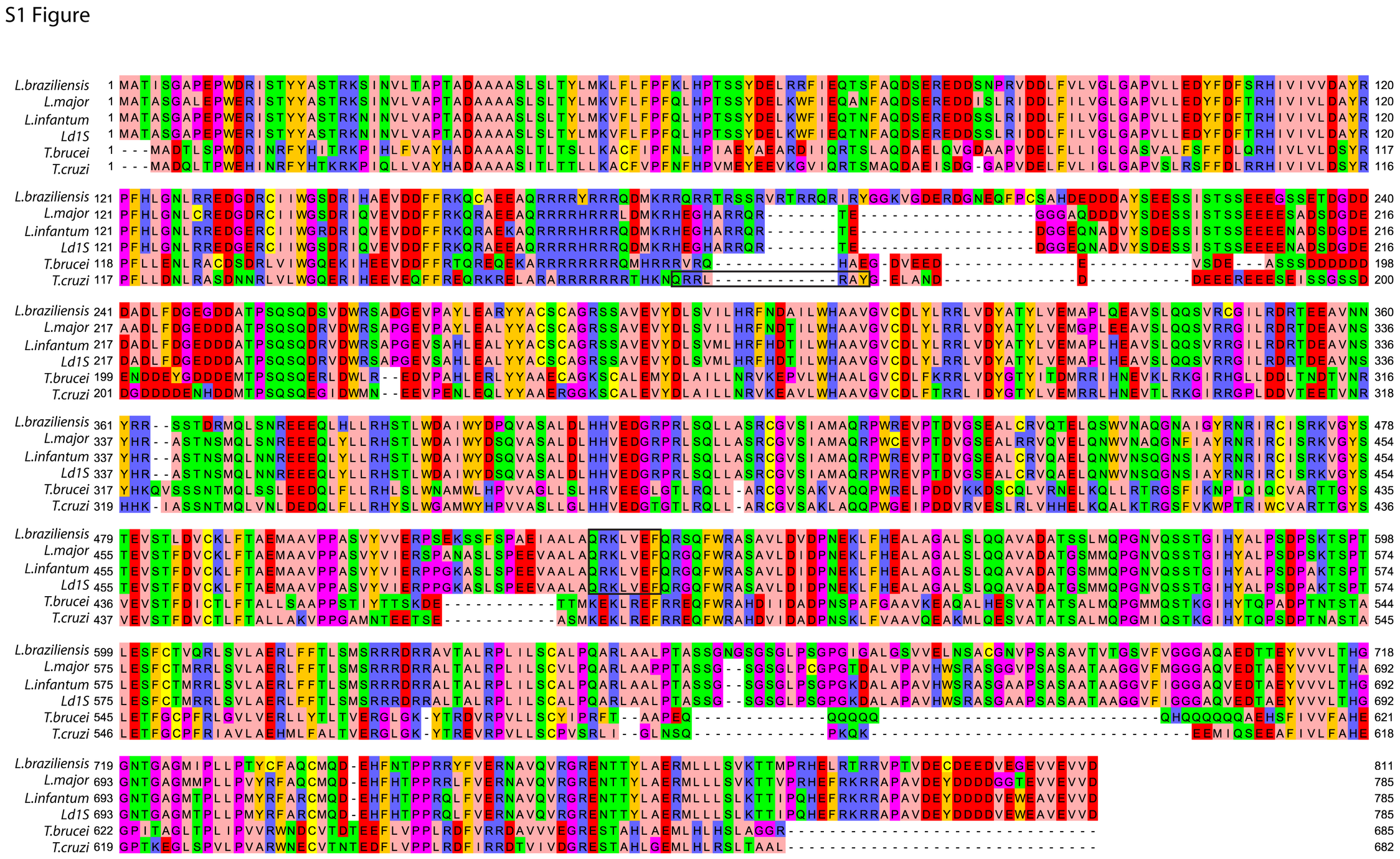

Supplement: S1 Fig — Clustal Omega analysis of LdCdc45 with Cdc45 of other trypanosomatids viewed using Jalview multiple alignment editor [7]. Black rectangles mark PIP boxes. Colours are indicative of the physico-chemical properties of the amino acids. Pink, aliphatic/hydrophobic; orange/ochre, aromatic; purple, glycine/proline; dark blue, basic; green, hydrophilic; red, acidic; yellow, cysteine. (TIF) [file ppat.1008190.s002.tif]

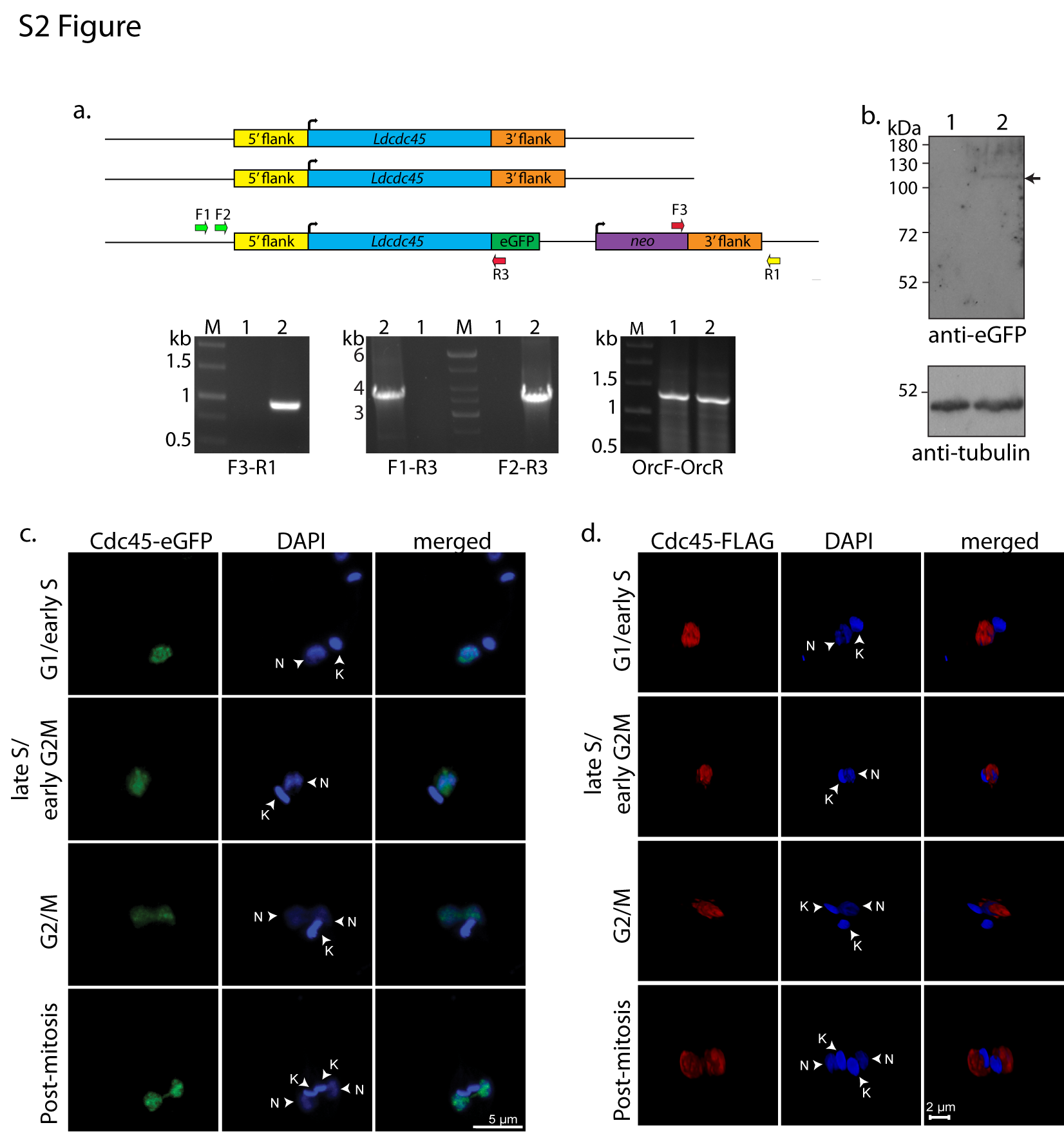

Supplement: S2 Fig — a. Tagging one cdc45 genomic allele with eGFP. Primers used in screening indicated by arrows. Agarose gels depict screening across replacement junctions, with primer pairs marked below. Lanes 1 –Ld1S, lanes 2 –replacement line. b. Western blot analysis of whole cell lysates probed with anti-eGFP antibodies (already available in the lab). Lane 1 –Ld1S, lane 2 –replacement line. c. Immunofluorescence analysis of Cdc45-eGFP at different cell cycle stages using kinetoplast morphology and number as cell cycle stage marker. d. Immunofluorescence analysis of Cdc45-FLAG at different cell cycle stages using kinetoplast morphology and number as cell cycle stage marker. G1/early S: one nucleus, one short or roundish kinetoplast; late S/early G2/M: one nucleus, one elongated kinetoplast; G2/M: two nuclei, one kinetoplast or one nucleus, two kinetoplasts; post-mitosis: two nuclei, two kinetoplasts. (TIF) [file ppat.1008190.s003.tif]

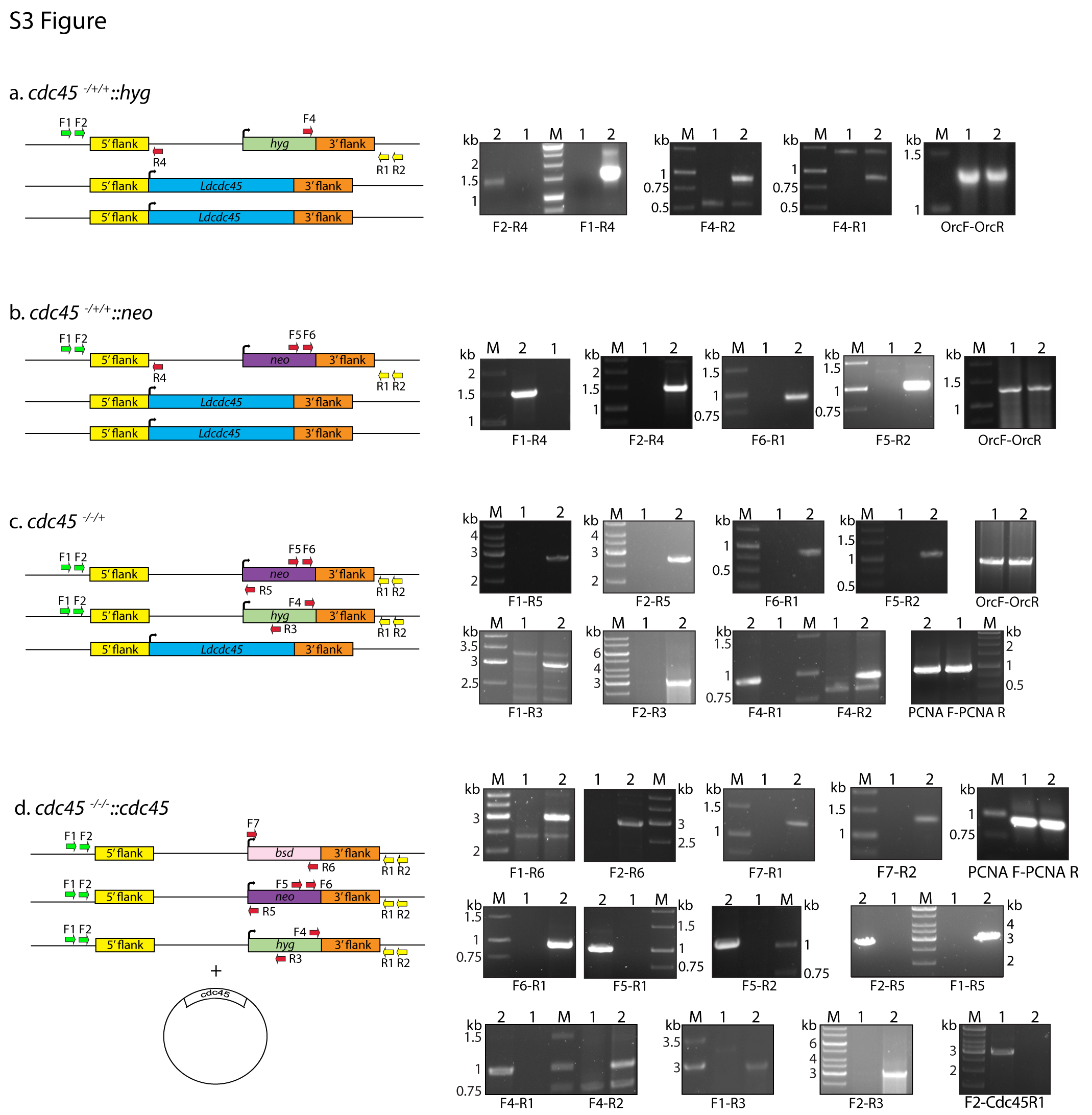

Supplement: S3 Fig — a: Replacement of one genomic allele with hygr cassette. b. Replacement of one genomic allele with neor cassette. c. Replacement of second genomic allele in cdc45-/+/+::hyg with neor cassette. d. Replacement of third genomic allele in cdc45-/-/+::cdc45 strain with bsdr cassette. Line diagrams represent schematics of knockout lines created. Primers used in screening are indicated in the line diagrams. Agarose gels depict screening of knockout lines. Primer pairs used in each case are indicated below the gel. Lanes 1- Ld1S, lanes 2- respective knockout line, M- DNA ladder marker. OrcF-OrcR and PCNAF-PCNAR PCRs served as positive controls. (TIF) [file ppat.1008190.s004.tif]

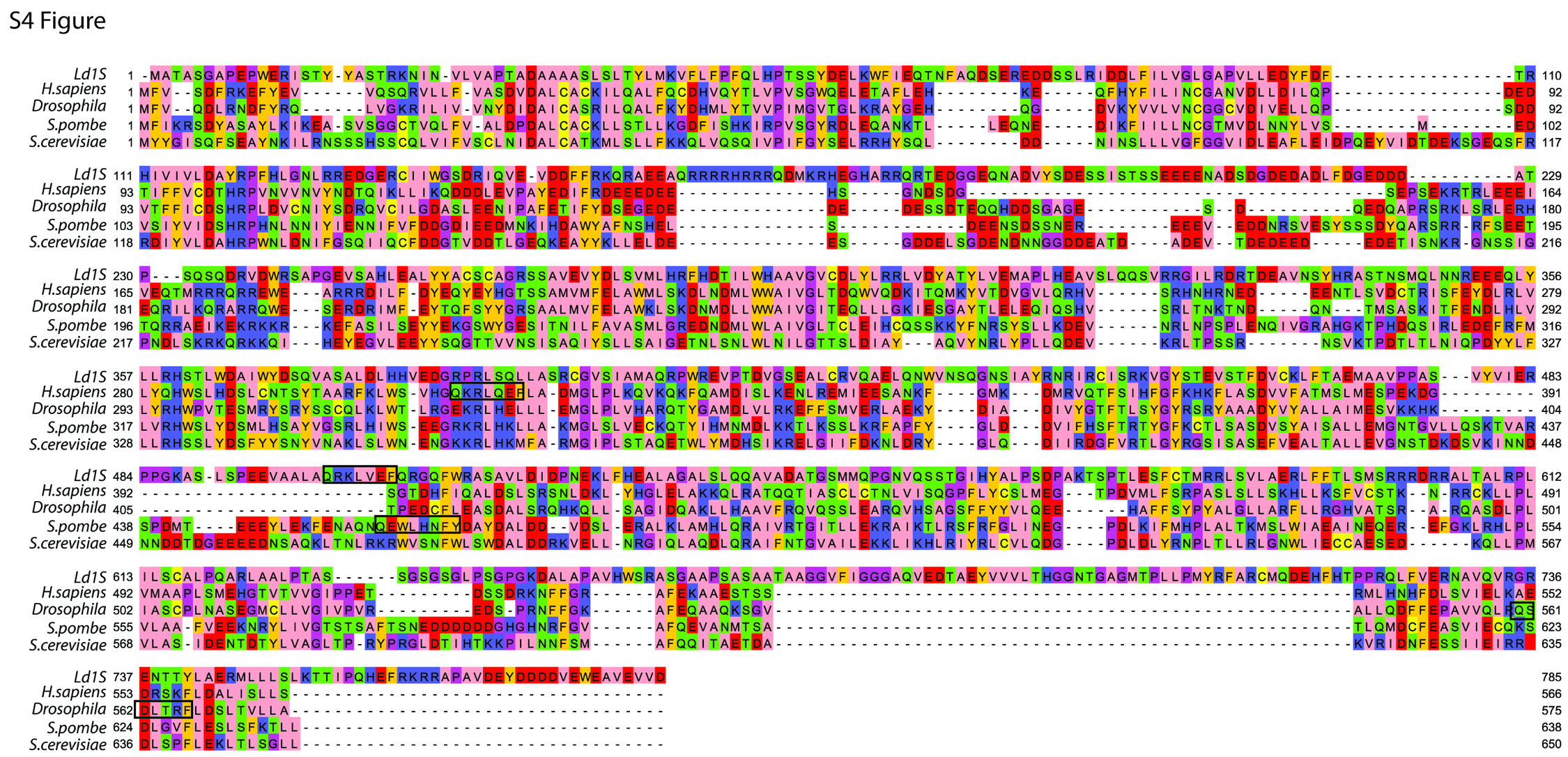

Supplement: S4 Fig — Clustal Omega analysis viewed using Jalview multiple alignment editor [7]. Black rectangles mark PIP boxes. Colours indicative of physico-chemical properties of the residues. Pink, aliphatic/hydrophobic; orange/ochre, aromatic; purple, glycine/proline; dark blue, basic; green, hydrophilic; red, acidic; yellow, cysteine. (TIF) [file ppat.1008190.s005.tif]

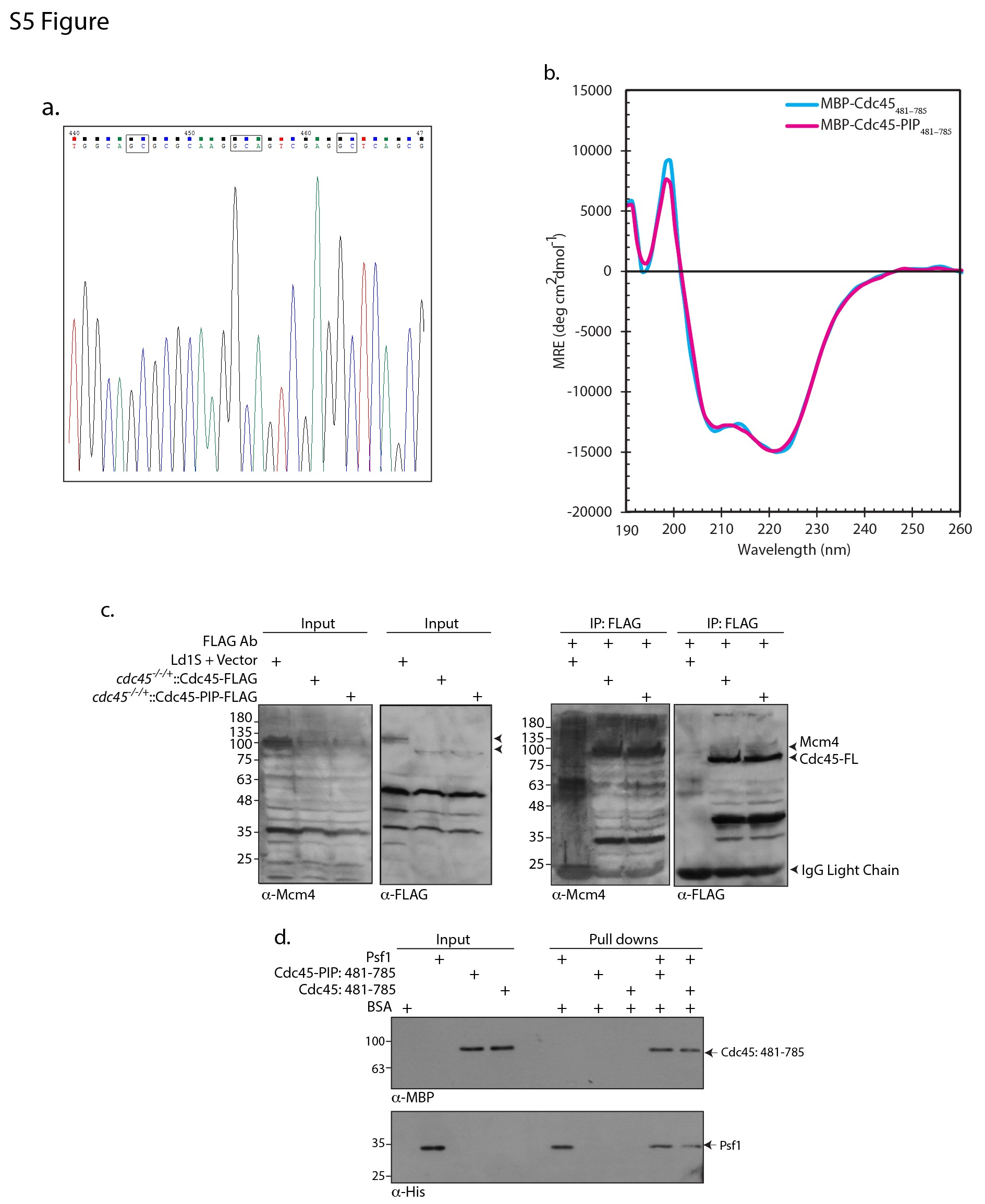

Supplement: S5 Fig — a. Confirmation of PIP box mutations by sequencing. Boxed residues indicate mutated nucleotides. b. CD spectra of MBP-Cdc45481-785 and MBP-Cdc45-PIP481-785 are depicted as a measure of mean residue ellipticity. c. Analysis of Cdc45-FLAG and Cdc45-PIP-FLAG immunoprecipitates from lysates isolated from transfectant cdc45-/-/+ cells. Western blot analysis done using mouse anti-Mcm4 antibodies (previously raised in the lab [5], 1:500) and mouse anti-FLAG antibodies (Sigma, 1:1000). The blots were first probed with anti-Mcm4 antibodies, and then the same blots were probed with anti-FLAG antibodies, due to which traces of the MCM4 protein (98 kDa) are also visible on the anti-FLAG blots (Cdc45-FLAG size 87 kDa). d. Analysis of pull-down reaction between MBP-Cdc45481-785 and LdPsf1, and MBP-Cdc45-PIP481-785 and LdPsf1. Western blot analysis was done using anti-MBP (Sigma, 1:12000) and anti-His (Sigma, 1:5000) antibodies. The experiment was done twice with comparable results; results of one experiment are shown. (TIF) [file ppat.1008190.s006.tif]

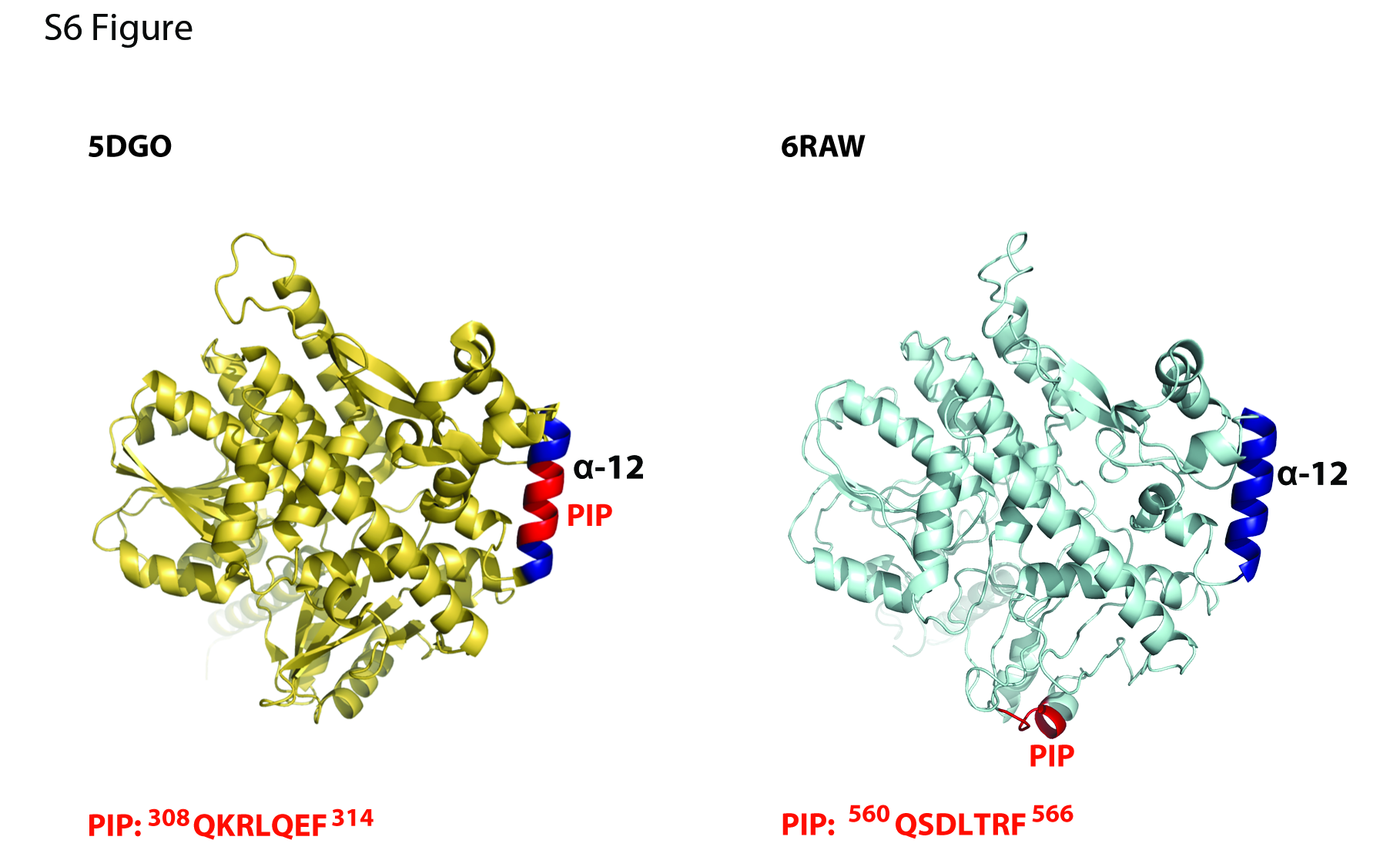

Supplement: S6 Fig — Left panel: Image of human Cdc45 derived from crystal structure PDB ID: 5DGO [8]. Navy blue region: α12 helix. Red region: PIP box, sequence below structure. Right panel: Image of Drosophila Cdc45 derived from electron microscopy structure PDB ID: 6RAW [9]. Navy blue region: α12 helix. Red region: PIP box, sequence below structure. (TIF) [file ppat.1008190.s007.tif]
