# Supplementary material for: Correction: DNA replication protein Cdc45 directly interacts with PCNA via its PIP box in Leishmania donovani and the Cdc45 PIP box is essential for cell survival
Source: PLoS Pathog. 2023 Mar 31;19(3):e1011303. doi: 10.1371/journal.ppat.1011303 (PMC10065266; doi:10.1371/journal.ppat.1011303)
Supplement: S1 File — Contains descriptions of various methods used in the study. (DOCX) [file ppat.1011303.s001.docx]

**Supplementary Methods:**

***Manipulations of Leishmania promastigotes***

Whole cell extracts were prepared from *Leishmania donovani* 1S promastigotes using M-PER reagent (ThermoFisher Scientific, USA). Growth patterns and generation time were analyzed as described earlier [1]. Promastigotes were synchronized using 5 mM hydroxyurea as previously, with the drugs (hygromycin/G418/bleomycin/blasticidin) being withdrawn 72 hours prior to setting up hydroxyurea-induced blocks [2,3]. Cells were fixed at different time-points after release from block, stained with propidium iodide, and analyzed by flow cytometry as described [2]. Transfections of *Leishmania* promastigotes and creation and maintenance of clonal lines were done as earlier [3].

***Manipulations using Schizosaccharomyces pombe***

Transformations of *sna41^goa1^* cells (kind gift from Prof. Hisao Masai, Tokyo Metropolitan Institute of Medical Science) were done by the lithium acetate method detailed in the Fission Yeast Handbook of the Nurse Lab hosted on <https://wolflab.squarespace.com/protocols/>, and transformants were selected for by plating on EMM2 medium lacking leucine, as the transforming plasmid carried the *leu* marker. Isolation of whole cell lysates was done by a RIPA-based method as described in the Fission Yeast Handbook of the Nurse Lab. To carry out complementation assays, cells (wild type, *sna41^goa1^* or transformants) were grown in EMM2 plus the necessary supplements to stationary phase at 25°C, then seeded at OD_595_ = 0.1 and allowed to grow at the same temperature till OD_595_ = 0.4. The cultures were then streaked out (10 μl of each culture) and the plates incubated at 25°C, 30°C or 37°C for two to four days before imaging.

***Cloning of Leishmania donovani cdc45 and psf1 genes***

The *cdc45* gene was amplified off Ld1S genomic DNA using Phu DNA polymerase and end primers designed against the *cdc45* gene sequence from the *Leishmania donovani* strain BPK282A1 genome (TriTrypDB ID: LdBPK_332450.1, [4]; primers used were Cdc45-FLAG-F: 5’-CACCGCAGGATCCACCATGGCGACAGCTTCC-3’ and Cdc45-FLAG-R: 5’-TCGATATCGTAGTCCACCACCTCCAC-3’), and the amplicon cloned into the pENTR/D-TOPO vector (Invitrogen) for sequencing.

For expression in *Leishmania* promastigotes the gene was resected from the pENTR clone using BamHI-EcoRV digestion and inserted into the corresponding sites in the pXG-/GFP+/FLAG vector [5] as well as pXG(*bleo*)/FLAG vector [6], to generate plasmids pXG/Cdc45-FLAG and pXG(*bleo*)/Cdc45-FLAG respectively. The *cdc45 –PIP* (mutated) gene was constructed by overlap PCR. The N-terminal part of the gene was amplified using the Cdc45-FLAG-F primer in combination with the Cdc45-PIP-R primer carrying the mutated sequence (5’-CCGCTGA**GC**CTCGAC**TGC**CTTGCGC**GC**TGCCAACGCCGCC -3’). The C-terminal part of the gene was amplified using the Cdc45-PIP-F primer carrying the mutated sequence (5’-TTGGCA**GC**GCGCAAG**GCA**GTCGAG**GC**TCAGCGG-3’) in combination with the Cdc45-FLAG-R primer. The two amplicons were then mixed and used as template in a PCR with primers Cdc45-FLAG-F and Cdc45-FLAG-R to obtain the full length *cdc45-PIP* gene. The amplicon was cloned into the BamHI-EcoRV sites of pXG-/GFP+/FLAG and pXG(*bleo*)/FLAG vectors, to generate plasmids pXG/Cdc45-PIP-FLAG and pXG(*bleo*)/Cdc45-PIP-FLAG respectively. The entire amplicon was sequenced to confirm the veracity of the mutated gene.

For expression of Cdc45_481-785_ and Cdc45-PIP_481-785_ in *E.coli,* the truncated genes (wild type or PIP mutant) were amplified using primers Cdc45Δ1-480-F (5’-TCGGATCCATGATCGAGAGGCCCCCAGGGA-3’) and Cdc45-R (5’-TCGTCGACG AATTCGCGGCCGCCTAGTCCACCACCTCCAC-3’), the amplicons cloned into the SmaI site of pUC18, and then resected using BamHI-SalI digestion followed by insertion into the corresponding sites in vector pMAL-c2X (NEB), to create plasmids pMAL/Cdc45Δ1-480 and pMAL/Cdc45-PIPΔ1-480.

For expression of Cdc45 (wild type or PIP mutant) in *Schizosaccharomyces pombe* the genes were amplified off their respective pXG/FLAG clones using the Cdc45-FLAG-F primer and a reverse primer corresponding to the region ~120 bp downstream of the FLAG tag in the pXG/FLAG vector (5’-TGCAGATGAACTTCAGGGTCAG-3’), and the amplicons were cloned into the SmaI site in the polylinker of the plasmid pART1 (a kind gift from Dr. Nimisha Sharma, Guru Gobind Singh Indraprastha University, New Delhi).

For expression of Psf1 in *E.coli* the gene was amplified off *Leishmania donovani* 1S genomic DNA using primers Psf1-F and Psf1-R (5’- TCTCGAATTCATGGGCTCC AAAAAACAGGATGC – 3’ and 5’- TCTCGGATCCGTGCCCCACCAGCGCGAC – 3’) and cloned into the SmaI site of pUC18, followed by resecting the insert using EcoRI and BamHI and cloning into the corresponding sites of pASK-IBA43plus (IBA BioTAGnology) to create plasmid pASK-Psf1. Psf1 thus expressed was His-tagged at its N-terminus and Strep-tagged at its C-terminus, and purified by Strep-Tactin II chromatography.

***Cloning of Schizosaccharomyces pombe cdc45 gene***

The *cdc45* gene of *Schizosaccharomyces pombe* was amplified off genomic DNA by overlap PCR, using the genome sequence of *S. pombe* available in the PomBase database ([www.pombase.org](http://www.pombase.org)) to design primers. The gene sequence encoding the N-terminal part of the protein was amplified using the primer pair SpCdc45-F (5’-TCGGATCCGTTATGTT CATCAAGAGATC-3’) and SpCdc45-exon1rev (5’-TTCTCGTTCTTGCTCATTAATAGC TTCAGCAAT-3’), while the sequence translating into C-terminal part of the protein was amplified using the primer pair SpCdc45-exon2for (5’-GCTGAAGCTATTAATGAGCA AGAACGAGAAT-3’) and SpCdc45-R (5’-TCGGATCCTTATAATAGTGTTTTGAAGGA CAG-3’). The full length gene was then cloned into pJET vector creating the construct pJET/SpCdc45. For expression of SpCdc45 protein in *sna41^goa1^* strain the gene was released from pJET/SpCdc45 using BamHI and subcloned into BamHI site of pART1 vector creating the construct pART1/SpCdc45.

The Sp*cdc45-PIP* mutant gene was created by overlap PCR, using the primer pair SpCdc45-F1 (5’-ATGTTCATCAAGAGATCCGATTAC-3’) and SpCdc45-R3-mut (5’-ATCATA**TGC**ATTGTG**GGC**CCATTC**TGC**ATTTTGAGCATTTTCAAAC-3’) to amplify the gene sequence encoding the N-terminal part of the protein, while the sequence translating into C-terminal part of the protein was amplified using the primer pair SpCdc45-F1mut (5’-GTTTGAAAATGCTCAAAAT**GCA**GAATGG**GCC**CACAAT**GCA**TATGAT-3’) and SpCdc45R1 (5’-TAGGATCCTTAGTGATGGTGATGGTGATGTAATAGTGTTTTGAA -3’). The full-length gene was then cloned into SmaI site of pUC vector, creating the construct pUC/SpCdc45-PIP. For expression of His-tagged SpCdc45-PIP protein in *sna41^goa1^* strain the gene was released from pUC/SpCdc45-PIP using BamHI-SacI and subcloned into BamHI-SacI sites of pART1 vector creating the construct pART1/SpCdc45-PIP.

***Tagging Cdc45 genomic allele with eGFP***

Genomic allele of *cdc45* was tagged with *eGFP* using homologous recombination. The donor plasmid for this was constructed by first amplifying the *cdc45* gene along with the ~ 0.82 kb region immediately upstream of it (using primers Cdc45-Rep-5’FL-F: 5’-CACCCCATGG**GATATC**GTGAGGGTACCGTTTGG-3’ and Cdc45-R1: 5’-TCCCAT GGATCCGTCCACCACCTCCACGG-3’) using genomic DNA as template, and cloning this amplicon into the NcoI site upstream of the *eGFP* gene in the pLEXSY_I-egfp-neo3 vector (Jena Bioscience) such that Cdc45 would be expressed in fusion with eGFP. This was followed by cloning the ~ 0.83 kb region immediately downstream of the *cdc45* gene (amplified off genomic DNA with primers Cdc45-Rep-3’FL-F: 5’-CACCACTAGTTGCGCGACACTGGACTTG-3’ and Cdc45-Rep-3’FL-R: 5’-TCACTAGT **GATATC**GCAGTTAGCGCGTTCCTT-3’) into the SpeI site adjacent to and downstream of the neomycin resistance cassette. The donor cassette was released from the plasmid so generated (pLEXSY-neo/ 5’FL-Cdc45/3’FL) by EcoRV digestion (site present in the primers defining the outer boundaries of the donor cassette), purified, and transfected into *Leishmania* cells. Clones were selected for on semi-solid M199 carrying G418 (50 μg/ml) as earlier [3], and screened by PCRs across the replacement junctions.

***Creating cdc45 knockout and rescue lines***

Cdc45 knockout lines were created by homologous recombination. To create the donor plasmids pCdc45-KO/neo and pCdc45-KO/hyg first the ~ 0.83 kb region immediately downstream of the *cdc45* gene (as above) was cloned into the SpeI site adjacent to and downstream of the neomycin/hygromycin resistance cassette in pLEXSY_I-egfp-neo3 and pLEXSY-eGFP/hyg [3] respectively. This was followed by amplification of the first 100 bp of *cdc45* along with the ~1kb region immediately upstream of the gene (using primers Cdc45-5’FL-F: 5’-TAGCGGCCGC**GATATC**AGTGTCGGCACTGCTATAGCA-3’ and Cdc45-R2: 5’-GCGGCCGCGTCGGCCGTT-3’) off genomic DNA, and cloning the amplicon into the NotI site of the vectors. EcoRV digestion was used to release the donor cassettes from these plasmid constructs (pCdc45-KO/neo and pCdc45-KO/hyg). To construct donor plasmid pCdc45-KO/bsd, the blasticidin resistance gene was amplified off plasmid pLPBLP (a kind gift from Dr. Aruna Naorem, University of Delhi South Campus) using the primer pair Bsd-F (5’-TCGGATCCATGGATCAATTTAACATTTCTC-3’) and Bsd-R (5’-TCACTAGTCTCGAGTTAATTTCGGGTATATT-3’), and BamHI-SpeI digestion used to insert this amplicon in place of the neomycin resistance cassette in pCdc45-KO/neo.

In creating Cdc45 knockout lines the donor cassettes from pCdc45-KO/hyg or pCdc45-KO/neo were transfected into *Leishmania* promastigotes and clones selected for using hygromycin (16 μg/ml) or G418 (50 μg/ml) respectively, as earlier [3]. The heterozygous knockouts thus obtained were named *cdc45*^-/+/+^::hyg or *cdc45*^-/+/+^::neo respectively. The *cdc45*^-/+/+^::hyg was transfected with the donor cassette from pCdc45-KO/neo and *cdc45*^-/-/+^ clones selected for using hygromycin and G418. Prior to attempting knockout of the third allele, the plasmid pXG(*bleo*)/Cdc45-FLAG was transfected into *cdc45*^-/-/+^ promastigotes and clones selected for on semi-solid M199 carrying G418, hygromycin, and bleomycin (2.5 μg/ml). A clone expressing Cdc45-FLAG robustly was used for further manipulations (*cdc45*^-/-/+^::Cdc45-FLAG). In attempting to knock out the third genomic allele of *cdc45* the donor cassette released from pCdc45-KO/bsd (by EcoRV digestion) was simultaneously transfected into *cdc45*^-/-/+^ and *cdc45*^-/-/+^::Cdc45-FLAG promastigotes. Clones were selected for using G418, hygromycin, and blasticidin (15 μg/ml) in case of the former, and using G418, hygromycin, bleomycin and blasticidin (15 μg/ml) in case of the latter.

The *cdc45*^-/-/+^::Cdc45-FLAG line was used in rescue experiments. For analyses of the role of the PIP box, plasmid pXG(*bleo*)/Cdc45-PIP-FLAG was transfected into *cdc45*^-/-/+^ promastigotes and clones selected for using G418, hygromycin and bleomycin. The clone obtained was named *cdc45*^-/-/+^::Cdc45-PIP-FLAG.

***Isolation of extracts from intracellular parasites***

J774A.1 cells were plated in 100 mm culture dishes (8x10^6^ cells per dish) in DMEM complete medium and incubated at 37°C in an atmosphere of 5%CO_2_ for 24 hours before infection with *Leishmania* metacylics at a 10:1 parasite:host ratio in serum-free medium at 37°C for 5 hours. The medium was aspirated and the dish rinsed with 1X PBS to remove uninternalized parasites, followed by refeeding with complete DMEM. The infected cells were incubated for 48 hours at 37°C, the medium aspirated, and the cells washed with chilled 1X PBS.

Cells were scraped in 10 ml chilled 1X PBS and collected in a tube for counting and harvesting. The cells were harvested by centrifugation at 1000*g* at 4°C for 10 minutes, resuspended in 50 μl RIPA buffer (50 mM Tris-Cl (pH 7.4), 150 mM NaCl, 0.1%SDS, 1% sodium deoxycholate, 1% NP-40, 1 mM EDTA) with protease inhibitor cocktail for lysis, and incubated on ice for 10 min with intermittent mixing. This was followed by three snap-freeze/thaw cycles in liquid nitrogen and ice-cold water before high speed centrifugation at 4°C for 20 min to clarify the lysate. The clarified lysate was analyzed by western blotting using *Leishmania* anti-PCNA antibodies which do not cross-react with human PCNA to ensure intracellular parasite proteins were isolated. This was followed by probing for Cdc45-FLAG.

***Immunofluorescence analysis***

The expression of Cdc45-eGFP was microscopically analyzed by observing eGFP fluorescence indirectly. For this asynchronously growing cells were fixed in 2% PFA, spread on poly-lysine coated coverslips for adherence, permeabilized with 0.1% TX-100, and probed with anti-eGFP antibodies (already available in the lab, mouse, 1:100 dil) for 2 h at room temperature before washing off excess antibody. This was followed by incubation with FITC-labelled secondary antibody (Jackson Immunoresearch Laboratories, 1:200 dil), washing away of excess antibody, and mounting in DAPI-containing Vectashield solution (Vector Laboratories). Images were taken using a confocal microscope with a 100X (oil) objective (LeicaTCS SP5 microscope, Leica LAS AF software). For observing Cdc45-FLAG expression, anti-FLAG antibody (Sigma Aldrich) was used at 1:100 dilution and Texas Red-labeled mouse secondary antibody (Jackson Immunoresearch Laboratories) was used at 1:100 dilution. Following mounting in DAPI-containing Vectashield solution, images were taken using a Leica SP8 Falcon confocal microscope with LAX 3D reconstruction module.

***Purification of MBP-Cdc45_481-785_ proteins***

Cells expressing MBP or MBP-Cdc45_481-785_ proteins were harvested from 20 ml *E. coli* cultures harboring overexpressed recombinant proteins and resuspended in lysis buffer (100 mM Tris.Cl (pH 8), 150 mM NaCl, 1 mM EDTA, 10 mM β-ME, 10% glycerol) with lysozyme (1mg/ml) and protease inhibitors cocktail. The suspension was incubated on ice for 30 minutes followed by sonication. The lysate was clarified by high speed centrifugation and the clarified cell lysate loaded onto amylose resin (200 μl; New England Biolabs). The column was washed extensively with 100 mM Tris.Cl (pH 8), 1M NaCl, 1 mM EDTA, 10 mM β-ME, 10% glycerol. The bound proteins were eluted using 10 mM maltose in 100 mM Tris.Cl (pH 8), 200 mM NaCl, 1 mM EDTA, 10 mM β-ME, 10% glycerol.

***CD spectroscopy analysis***

MBP-Cdc45**_481-785_**  and MBP-Cdc45-PIP**_481-785_**  protein samples (75 µg) were prepared in 1X PBS and their CD spectra were recorded using a Jasco J-815 spectropolarimeter. Data was recorded from 260 to 190 nm in 1 nm steps (using a scanning speed of 200 nm/min at 25°C) using a cuvette of path length 0.1 cm. An average of 20 scans was used to plot each spectrum showing mean residue ellipticity, expressed as degrees cm^2^ dmol^-1^.

**References:**

1. Chandra U, Yadav A, Kumar D, Saha S (2017) Cell cycle stage-specific transcriptional activation of cyclins mediated by HAT2-dependent H4K10 acetylation of promoters in Leishmania donovani. PLoS Pathog 13: e1006615.

2. Minocha N, Kumar D, Rajanala K, Saha S (2011) Kinetoplast morphology and segregation pattern as a marker for cell cycle progression in Leishmania donovani. J Eukaryot Microbiol 58: 249-253.

3. Yadav A, Chandra U, Saha S (2016) Histone acetyltransferase HAT4 modulates navigation across G2/M and re-entry into G1 in Leishmania donovani. Sci Rep 6: 27510.

4. Aslett M, Aurrecoechea C, Berriman M, Brestelli J, Brunk BP, et al. (2010) TriTrypDB: a functional genomic resource for the Trypanosomatidae. Nucleic Acids Res 38: D457-462.

5. Minocha N, Kumar D, Rajanala K, Saha S (2011) Characterization of Leishmania donovani MCM4: expression patterns and interaction with PCNA. PLoS One 6: e23107.

6. Kumar D, Saha S (2015) HAT3-mediated acetylation of PCNA precedes PCNA monoubiquitination following exposure to UV radiation in Leishmania donovani. Nucleic Acids Res 43: 5423-5441.

7. Sievers F, Higgins DG (2014) Clustal Omega, accurate alignment of very large numbers of sequences. Methods Mol Biol 1079: 105-116.

8. Simon AC, Sannino V, Costanzo V, Pellegrini L (2016) Structure of human Cdc45 and implications for CMG helicase function. Nat Commun 7: 11638.

9. Eickhoff P, Kose HB, Martino F, Petojevic T, Abid Ali F, et al. (2019) Molecular Basis for ATP-Hydrolysis-Driven DNA Translocation by the CMG Helicase of the Eukaryotic Replisome. Cell Rep 28: 2673-2688 e2678.
